# Study protocol for an evaluability assessment of an anti-human trafficking program

**DOI:** 10.1186/s12939-021-01573-5

**Published:** 2021-10-26

**Authors:** Christopher J. Wretman, Rebecca J. Macy, Amanda M. Stylianou, Anita S. Teekah, Elizabeth N. Ebright, Jeongsuk Kim, Jia Luo, Cynthia Fraga Rizo

**Affiliations:** 1 The Cecil G. Sheps Center for Health Services Research, University of North Carolina at Chapel Hill, 725 M.L.K. Jr Blvd., Chapel Hill, NC 27516 USA; 2 School of Social Work, University of North Carolina at Chapel Hill, 325 Pittsboro Street CB #3550, Chapel Hill, NC 27599 USA; 3 Easterseals New Jersey, East Brunswick, NJ USA; 4 Safe Horizon, New York City, NY USA

**Keywords:** Study protocol, Evaluability assessment, Human trafficking, Global health

## Abstract

**Background:**

Human trafficking is a serious global challenge associated with a complex array of health inequities for individuals, families, and communities. Consequently, in addition to a conventional criminal justice approach, anti-trafficking scholars have increasingly called for a public health approach to address this global challenge. Such calls have emphasized that a comprehensive, robust, and social justice-informed public health strategy for anti-trafficking must include services to facilitate survivors’ HT exit and recovery, and to prevent their re-victimization. Fortunately, many community-based organizations and non-governmental organizations worldwide have heeded these calls and developed anti-trafficking programs for survivors. Unfortunately, despite the growing numbers of organizations providing anti-trafficking services, research concerning these programs’ effectiveness remains nascent overall, and even more scant when filtered through an equity focus.

**Methods:**

To advance the field by developing guidance concerning how best to evaluate anti-human trafficking programs, an ongoing research project aims to conduct a mixed methods evaluability assessment of a prominent anti-trafficking program using a social justice framework. Guided by well-established evaluability assessment frameworks, the study activities include four sequential steps: (a) focusing the assessment, (b) developing the program theory and logic, (c) gathering feedback, and (d) applying the assessment findings. Activities will include qualitative interviews and focus groups, observations, and quantitative analysis of program data among others. Human subjects and ethical review for the evaluability assessment has been granted by the Office of Human Subjects Research at The University of North Carolina at Chapel Hill.

**Discussion:**

Once completed, evaluability assessment results will provide evidence and products that have the potential to guide both evaluation research and service provision not only for the specific organization under study, but also for other anti-human trafficking programs worldwide. Findings will be developed into a variety of dissemination products tailored for both practice professionals and researchers. In the interim, this protocol manuscript offers research strategies and recommendations that can help inform the development of other studies in the developing field of anti-trafficking program evaluation research.

## Background

Human trafficking (HT) for labor and commercial sex is a serious global challenge associated with a complex array of detrimental health outcomes for individuals, families, and communities [1–4]. Research documenting HT survivors’ well-being has consistently demonstrated that HT-related experiences are profoundly traumatizing and likewise associated with significant injury in addition to serious mental health and physical illnesses [5–9]. Consequently, in addition to a conventional criminal justice approach, anti-trafficking scholars have increasingly called for a public health approach to address this global challenge [1–3]. Such calls for a public health approach complement the growing emphasis in healthcare systems on the social determinants of health (i.e., economic, living, social, and working conditions that can diminish or enhance health) as well as health equity (i.e., when all people can live their healthiest lives regardless of their economic, living, social, and working conditions) [10, 11]. Given the very nature of HT, which entails the use of force, fraud, or coercion to compel a person into commercial sex acts, labor, or services against their will, HT is a profound violation of human rights that often occurs to those without economic, personal, and social resources. Moreover, once HT occurs, victims may often be left with fewer resources, as well as with new problems stemming from the trauma of trafficking experiences [3, 12]. For all these reasons, a comprehensive, robust, public health approach to anti-trafficking must attend to equity by including services to facilitate survivors’ exit from trafficking, as well as to prevent their re-victimization [6, 7, 13, 14].

Fortunately, many community-based organizations and non-governmental organizations worldwide have heeded these calls and developed anti-trafficking programs for survivors. One organization engaged in this effort is Safe Horizon (SH), which is the largest non-profit provider of services for crime victims in the United States. With a staff of more than 1000 professionals, SH offers an array of services through a network of more than 100 program locations across the New York City area, directly working with more than 250,000 children, adults, and families affected by crime and abuse each year. Among many key initiatives, the Anti-Trafficking Program (ATP) at SH is a cornerstone of the organization and represents one of the largest providers of HT services in North America. Since its launch in 2001, ATP has worked with more than 1000 HT survivors from more than 80 countries.

Notably, however, ATP has never been externally evaluated. Far from being an outlier, SH is like many other service providers in not having rigorously evaluated its anti-trafficking program. Although there are growing numbers of organizations providing anti-trafficking services, research concerning these programs’ effectiveness remains nascent [2]. Recent reviews have underscored the field’s formative nature by revealing a dearth of studies focused on HT program evaluation, as well as highlighting critical knowledge gaps regarding the effectiveness of these programs [7, 15, 16]. In short, although there is currently a great deal of *effort* involved in developing and delivering anti-trafficking programs, there remains little *evidence* about these programs’ effectiveness. The limited state of evidence is worrisome because, without evidence of program effectiveness, well-meaning service providers run the risk of unintentionally do more harm than good as they seek to serve survivors [13]. Relatedly, given the extent of HT survivors’ needs and vulnerabilities, conducting evaluations in the complex and dynamic organizational settings in which survivors typically receive anti-trafficking services is not easily achieved. For all these reasons, the field could not only benefit from program evaluation research findings in themselves, but also from guidance concerning how best to carry out evaluation research of anti-trafficking programs [1, 2].

To help address these gaps, this paper outlines an ongoing research project funded under the auspices of the National Institute of Justice (#2019-VT-BX-0039). The project is comprised of two distinct and sequential study phases. The first, which aims to conduct an evaluability assessment (EA) of ATP, began in August 2020 and will continue through July 2021. The second study phase will be conducted from 2021 to 2022 and will be a comprehensive formative evaluation of ATP. This protocol manuscript focuses on the EA, which is the first study phase, and which is a well-established research approach that has been successfully used to advance health policies and programs previously [17–19]. Moreover, given the nascent state of program evaluation research in the anti-trafficking field, the first phase of this project (i.e., the EA) was deemed critical to ensure that the study protocols developed for the second phase would be meaningfully, rigorous, and survivor-centered [20, 21].

### Aims

Specifically, this protocol outlines procedures of an EA study of SH’s ATP to develop program and research materials [17–19, 22]. This study has two specific aims:

- To determine the components of ATP as it is delivered in practice, including the program’s theory of change, implementation guide, logic model, and fidelity instruments (program materials).
- To determine the best approaches, methods, and measures to evaluate ATP rigorously and meaningfully (research materials).

## Methods/Design

### Setting

ATP at SH is a comprehensive HT survivor direct services program with four key components: (a) legal and immigration relief, (b) case management and counseling, (c) training and outreach, and (d) policy and advocacy. Overall, the program aims to enhance survivors’ autonomy, independence, self-sufficiency, quality of life, and well-being. ATP uses a comprehensive case management approach to achieve this aim via a range of services including (a) access to benefits and immigration remedies, (b) linkage to shelter, (c) trauma-informed counseling, (d) criminal justice advocacy, (e) legal referrals, and (f) vocational trainings and educational opportunities.

Overall, ATP provides an ideal setting to conduct an EA of a HT victim service program for three reasons. First, with 19 years of service delivery to trafficking survivors, ATP is an established and robust program that has demonstrated durability within New York City and surrounding communities. Second, ATP annually serves a sizable number of new survivors and routinely engages with a substantial number of survivors across multiple points in time, indicating strong potential for robust samples of survivor research participants. Third, ATP serves diverse survivors who have experienced labor, sex and/or forced marriage HT, regardless of background or socio-demographic characteristics.

### Personnel

The first key group of study personnel is the study team itself. The study team is divided into two distinct yet collaborative groups. The practice team, centered at SH in New York City, is comprised of SH staff who are anti-trafficking experts with extensive practice-based experience related to the development, delivery, and evaluation of services for HT survivors. The practice team’s primary responsibility is to facilitate survivor-focused study activities, and to provide expertise and knowledge regarding the ATP program itself. The research team, centered at The University of North Carolina at Chapel Hill, is comprised of experts with extensive research experience in qualitative and quantitative methods related to the evaluation of services for trafficking and violence survivors. The research team’s primary responsibility is to facilitate the research components of the study, including the collection, management, and analysis of all data.

A second key group of study personnel will be a Survivor Expert Advisory Group (S-EAG). These study personnel will be survivor-consultants who are alumni or current clients of the ATP program. The impetus for forming the S-EAG follows from the desire to center survivors’ lived experiences in the research, and also from recommended guidelines for collaborating with HT survivors that call on researchers to formally recognize survivors’ expertise, as well as to ensure their active contributions to studies [21, 23]. The rationale for such calls is built on three principles that will also guide the entire study: (a) conceptualize survivors’ lived experience as valuable expertise, (b) reduce the burden of research participation for survivors, and (c) ensure that research activities do not exploit survivor participants. S-EAG members will be recruited from ATP’s *Voices of Hope* survivor leadership group, word-of-mouth snowball sampling, and recruitment flyers. In addition, and consistent to the equity approach that will guide this project, the practice and research teams will use the following strategies to help ensure that survivors– no matter their backgrounds, characteristics, and situations– are aware of and able to participate in the advisory group. Specifically, the teams will (1) translate S-EAG recruitment materials into multiple languages (e.g., English, Spanish, Tagalog) and formats (e.g., email, text, .jpeg, .pdf); (2) acquire an on-demand phone interpretation service to use during recruitment and meetings; (3) offer various modes of communication to participants (e.g., text, emails, phone, Google Voice) throughout their participation in the advisory group to accommodate their communication preferences; (4) offer choice about virtual platforms for meetings (e.g., phone, Whatsapp, Zoom) to accommodate their participation preferences; (5) and offer meetings in the evening and on weekends as needed to accommodate their scheduling preferences. Overall, it is expected that approximately 10 survivor experts will participate in the S-EAG.

The final group of study personnel will be members of an Interdisciplinary Expert Advisory Group (I-EAG) comprised of practitioners and researchers with specific expertise in HT, community-engaged research, criminal justice responses to trafficking, evaluation, outcome instruments, public health and health equity, victim services, and trauma-informed care. The I-EAG will be comprised of 15 members selected prior to study funding who will provide feedback on all aspects of the project to ensure that project findings are meaningful and hold implications for the wider field. In addition to considerations of the I-EAG members’ expertise, the practice and research teams will use two strategies to ensure that the formation of is consistent with a health equity approach. First, before invitation are made to prospective I-EAG members, the practice and research teams will collaborate to generate a list of potential members who are professionally situated in advocacy, practice, and policy organizations, as well as in research organizations and universities. Likewise, the practice and research teams will consider invitees’ backgrounds, demographic characteristics, and intersecting identities in generating a list of prospective members.

### Approach

**Fig. 1:**
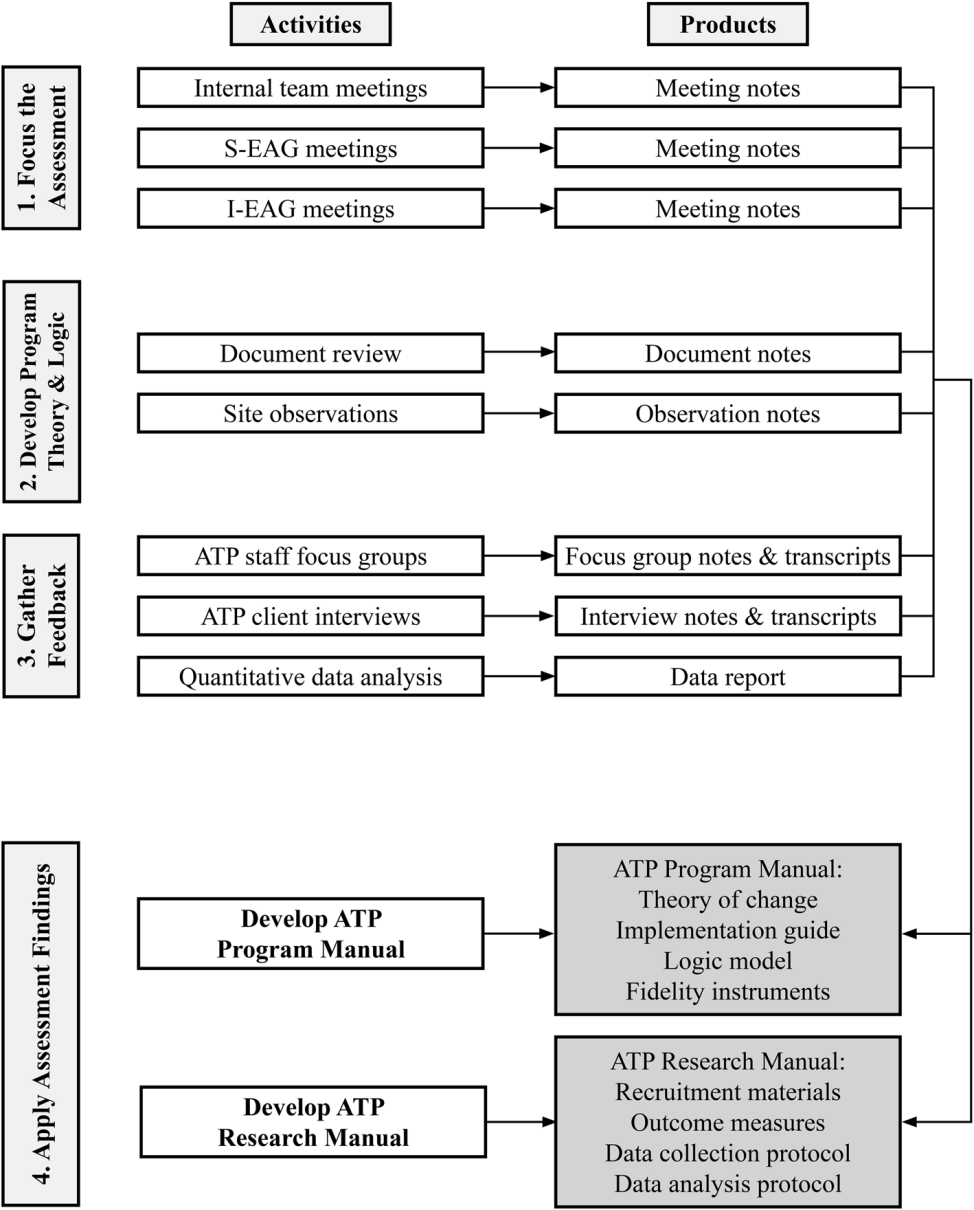
Framework of Study Steps, Activities, & Products. *Note*. S-EAG = Survivor Expert Advisory Group; I-EAG = Interdisciplinary Expert Advisory Group; ATP = Anti-Trafficking Program

As noted above, this study is an *evaluability assessment*—a noted methodological approach to evaluate and improve health programs that has gained increasing attention in recent years as a robust approach to foster social change as well as the development of practice-based evidence [18, 24, 25]. The study will be guided by Trevisan and Walser’s EA model and includes four sequential steps: (a) focusing the assessment, (b) developing the program theory and logic, (c) gathering feedback, and (d) applying the assessment findings that each have delineated activities and products (see Fig. 1) [19].

All study documents (e.g., protocols, data collection materials, data, and products) will be stored in a password-protected folder on the Microsoft Teams platform. Qualitative analyses will be conducted using NVivo and quantitative analyses will be conducted using Stata.

#### Step #1: focus the assessment

The first step involves clearly establishing the EA’s goals, scope, and boundaries, as well as developing initial assessment procedures. This step will consist of three activities: regular internal study team meetings, intermittent S-EAG meetings, and intermittent I-EAG meetings. These meetings are expected to elucidate key details related to the EA, including issues of development, implementation, and interpretation of all aspects of the study. All meetings will be conducted with a semi-structured agenda and, due to COVID-19 precautions, will be held via video conferencing.

#### Step #2: develop the initial Program Theory & Logic

The second step involves developing an initial program theory, logic model, and evaluation plan for ATP. To help to begin to elucidate how ATP is delivered in practice, this step will consist of two activities, document review and site observations, which are recommended strategies in the Trevisan and Walser’s EA model.

The document review will comprise a systematic review of all salient ATP and SH documents (e.g., mission statements, brochures, annual reports, grant proposals, webpages, meeting notes). Through a qualitative content analysis of these documents, which will be described subsequently, this activity will identify, categorize, and log key information about ATP and its provision of anti-trafficking services. Items to be collected will include all ATP documents that are both suitable for sharing with the research team (i.e., not protected nor confidential) and relevant to the vision, mission, and day-to-day activities of ATP (i.e., program-specific).

The site visits will have dual purposes of observing key ATP activities in action and developing relationships with key ATP stakeholders. Due to COVID-19 precautions, these observations will take place using video conferencing as opposed to in-person visits. The observations will target key ATP activities and meetings and the research team will follow the guidance of the practice team at SH to determine relevant activities to observe. Examples of meetings that will be observed include program planning meetings, regular staff meetings, and staff trainings. With the goal of further understanding how ATP operates and its programmatic goals, the project team developed a template for observational data collection to guide observers’ note taking and to facilitate future data analysis. The observation meetings will feature both (a) SH organizational leadership and (b) ATP program staff and will feature semi-structured agendas to facilitate conversation and learning. Meetings with SH leaders (*N* ≈ 5) will be designed to learn about the ATP’s (a) history and development, (b) overarching philosophy, (c) past and current funding streams, and (d), overall budget. Meetings with ATP staff (*N* ≈ 10) are expected to engender specific learning about macro-level program details such as (a) staff roles and responsibilities, (b) program culture and norms, and (c) staff perceptions of the project. Each observation/meeting will be documented by a team member using a standardized note form.

Two reviewers will independently analyze the program documents and the site visits notes using qualitative content analysis. Working both deductively (i.e., guided by intervention research frameworks) [26, 27] and inductively (i.e., allowing novel codes and themes concerning the program and program delivery to emerge from the analysis), the analysis processes will focus on determining the core components of ATP as well as how the program is delivered in practice to inform the development of initial ATP program materials. These initial versions of program materials will include draft components recommended by intervention research frameworks, including theory of change, implementation guide, logic model, and fidelity instruments [26–29]. Next, these initial program materials will be further developed, detailed, and finalized by findings from subsequent project steps as described below.

#### Step #3: gather feedback

The third step involves gathering feedback to inform the further development and refinement of the program’s logic model, theory of change, and evaluation plan. This step will consist of three activities: focus groups with ATP stakeholders, interviews with ATP clients, and quantitative analysis of ATP administrative data.

The stakeholder focus groups will include both ATP leaders (*N* ≈ 3) and ATP providers (*N* ≈ 8). The leadership focus group will include the ATP Senior Director, Supervising Attorney, and Supervising Social Worker. Meanwhile, the provider focus group will include staff members who conduct the day-to-day client and non-client work of ATP, including the social workers, staff attorneys, administrative staff, and training staff. All focus groups and interviews will be held via video conferencing and audio recorded for transcription purposes. The project team has developed standardized guides for both the focus groups and interviews that include guidelines, open-ended questions, and prompts. Broadly, the questions for stakeholders and survivors are grouped into sections relating to the following six topics: (a) experiences with the ATP, (b) perceptions of the importance of ATP services/components, (c) perceptions of meaningful ATP outcomes, (d) considerations of research with HT survivors, (e) considerations of research within the ATP, and (f) opinions on the impact of the COVID-19 global pandemic. Also, limited demographic information will be collected from survivors during the interviews. It is hoped that the qualitative data gathered from these focus groups and interviews will provide in-depth guidance regarding (a) core components and intervention activities, (b) intended outcomes, (c) existing data and protocols, and (d) preferences for future data collection and evaluation. Two or more reviewers will independently analyze the focus groups and interview transcriptions using qualitative content analysis.

**Fig. 2:**
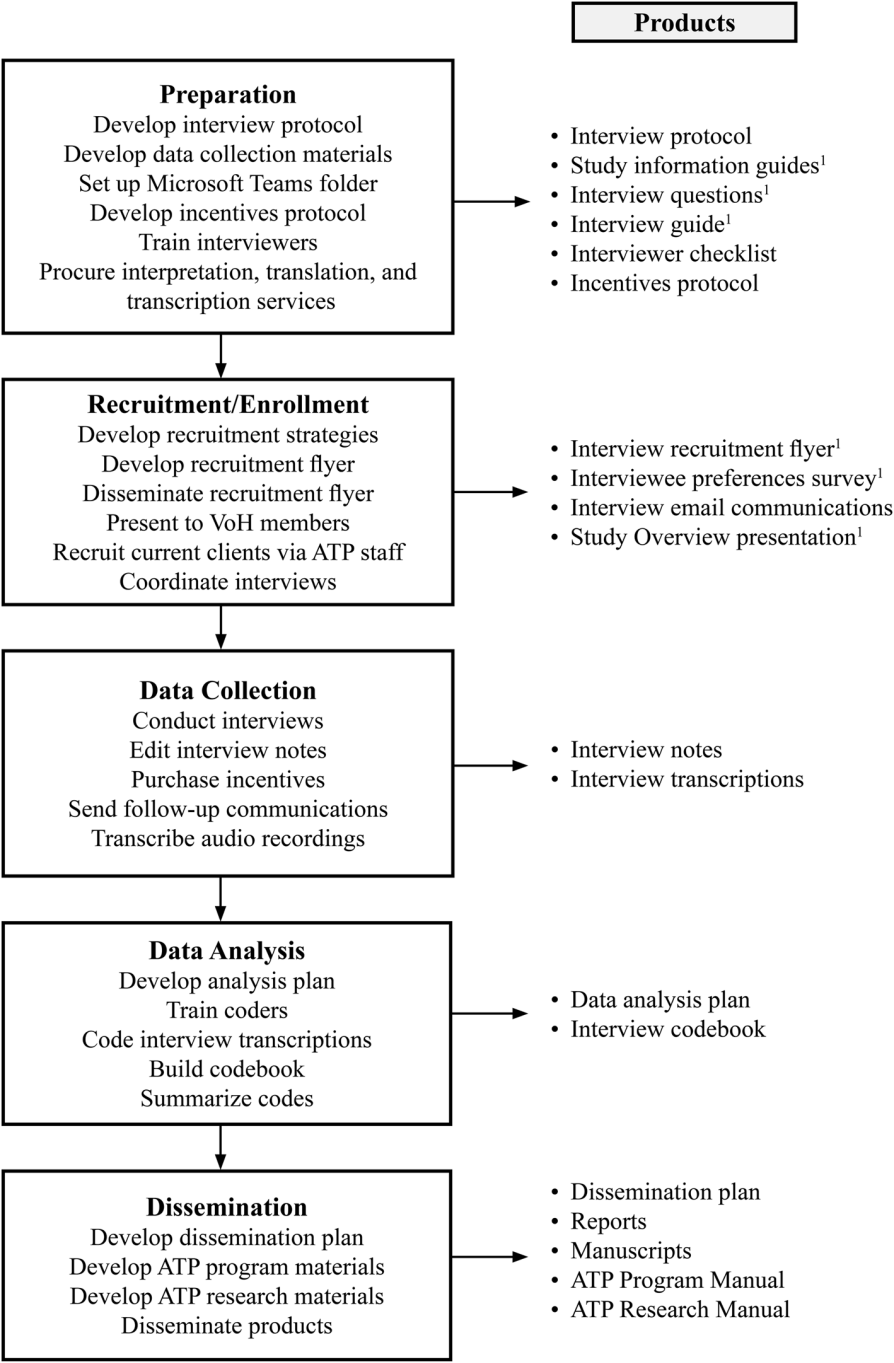
Survivor Interview Process & Products. *Note.* S-EAG = Survivor Expert Advisory Group; I-EAG = Interdisciplinary Expert Advisory Group; ATP = Anti-Trafficking Program; VoH = ATP *Voices of Hope* survivor leadership group. ^1^ Indicates that materials also translated into Spanish

The survivor interviews (see Fig. 2) will feature individuals (*N* ≈ 15–20) who are either current or former ATP clients. During the recruitment stage of the survivor interviews, potential participants will be emailed a study information guide and will be asked to provide verbal consent to be interviewed and to be audio recorded before the interview begins. The survivor interview guide for interviewers also lists specific pre- and post-interview action steps to ensure the safety and privacy of the participants. At the beginning of the interview, participants will be first asked, “Is now a safe time to talk?” and the interviewers will review the participant’s rights. Interviewers will also ask participants if it is necessary to determine a verbal signal in case the participant suddenly loses privacy during the interview. Following the interview, survivors will be provided with the option to informally debrief with an ATP staff member if desired. Interviewers will be instructed to contact an ATP staff member after the interview if they have safety-related concerns or if participants show signs of distress. To protect participant’s confidentiality, all data will be aggregated and summarized during the dissemination of findings.

Quantitative data analyses of administrative SH data will comprise analyses on observed extant data records on ATP participants past and present (*N* ≈ 500) within SH records as of June 2021 near the end of the EA. The goals of this activity are to determine the extent to which ATP’s logic model is consistent with the program as delivered and to inform the development of materials for subsequent research. Specifically, analyses will explore different service needs across subcategories of survivors including trafficking type, gender, international/national trafficking, and others. The variables in the data file will include information within three overarching domains related to (a) survivor demographic characteristics (e.g., country of origin, ethnicity/race, gender, primary language), (b) survivor trafficking experiences (e.g., type, victimization location, type of exit), and (c) survivor ATP service provision (e.g., type, frequency, duration of services accessed). All data will be de-identified and shared by the SH practice team for analysis by the research team.

#### Step 4: apply the assessment findings

The final step of the evaluability assessment will be to integrate and apply the findings from Step #2 (i.e., the document review and the site visits) as well as Step #3 (i.e., stakeholder focus groups and survivor interviews) to practice and research materials that can inform the future formative evaluation of ATP. This step will consist of two activities that will be guided by well-established intervention research frameworks [26–29], as well as Trevisan and Walser’s EA model. First, the team will develop a comprehensive *ATP Program Manual* containing four items: (a) theory of change, (b) implementation guide, (c) logic model, and (d) fidelity instruments. Second, the team will develop a comprehensive *ATP Research Manual* consisting of four items: (a) recruitment materials, (b) outcome measures, (c) data collection protocol, and (d) data analysis protocol. Both manuals will be custom-designed, full-color products available in print and electronic forms. In turn, these two manuals will guide and inform the second study phase, which will be a comprehensive formative evaluation of ATP to be conducted in 2021 and 2022.

### Mitigation of potential risks

Our team has identified potential pitfalls and challenges as well as strategies to ensure the project’s success. First, as experienced community-based researchers, we realize that some of the project’s assumptions might need to be modified. Fortunately, the EA model guiding our evaluability assessment is adaptive, flexible, and iterative [16–19]. Our team is experienced in conducting research in complex and dynamic settings, as well as adapting strategies to meet the needs of community partners and research participants, while ensuring project goals are met and findings are disseminated [30–35]. For example, due to the COVID-19 global pandemic, all of the in-person data collection efforts will have been shifted to virtual data collection, resulting in adaptations to the original data collection plan.

Our team will also track challenges, document problem-solving strategies related to these challenges, and note research successes. By documenting lessons learned, our team can help inform the broader anti-trafficking field on best practices about conducting research with HT survivors. Second, our team realizes that care and thought will need to be put into to ensuring successful recruitment to achieve our data collection goals while also safeguarding survivors’ confidentiality, protection, and well-being. Toward this end, our team’s plans are guided by successful research recruitment efforts from prior studies with survivor participants. Our team will also seek feedback from our expert advisory groups on our recruitment plans and make necessary enhancements before data collection.

### Statistical analysis

Statistical analysis will be required for the following study activities: (a) document review, (b) survivor interviews, and (c) analysis of administrative data.

Analyses for the document review activity will comprise calculation of univariate statistics (e.g., frequency, proportion, mean, standard deviation) to characterize the documents and the elements within them. Likewise, analyses of the demographic characteristics captured during the survivor interviews will comprise calculation of similar univariate statistics for the overall group of interviewed survivors. Such statistics will be important for characterizing the nature of the sample both during analysis as well as when findings are disseminated given the diversity of people who experience trafficking in terms of their background, demographics, and types of trafficking experiences.

Analysis of the administrative data will be more involved and complex. Primary analyses will comprise univariate descriptive statistics in addition to bivariate comparisons of differences using appropriate significance tests (e.g., Fisher’s exact tests, Wilcoxon rank-sum tests, one-way analysis of variance). Analyses will be conducted overall for all survivor observations in the data, and also on sub-groups of survivors based on the following four types of HT victimization: (a) sex trafficking only, (b) labor trafficking only, (c) sex and labor trafficking, and (d) other/unknown. The exact nature of the bivariate tests will be fully determined after data collection but are expected to feature tests of (a) type × demographic characteristics, (b) type × trafficking characteristics, and (c) type × service provision. The research team posits that there will be some significant results among these tests that will be instrumental in better understanding the survivors in the ATP program, and how the program itself can better provide tailored services. Also, as noted above, given the heterogeneity among those who experience trafficking, as well as those served by SH, such analyzes will help investigate how these anti-trafficking services work for survivors across diverse background, which is an important tenant of a health equity approach. All tests will be reported with point estimates, 95% confidence intervals, and *p* values. Secondarily, following from any significant bivariate tests the research team may choose to explore additional multivariable (a) ordinary linear and (b) generalized linear regression models. Such models could feature a variety of forms including potentially with (a) HT type as the dependent variable regressed on demographic characteristics, (b) trafficking experience as the dependent variables regressed upon demographic and/or trafficking characteristics, or (c) ATP service provision as the dependent variable regression on individual characteristics. The exact specification of the distributional family (e.g., Gaussian, binomial), link function (e.g., identity, logit), and effects (e.g., fixed, random) will be considered individually depending on the nature of dependent variable for each model.

### Statistical power

The study’s statistical power analyses are focused solely on the analysis of the ATP administrative data. Preliminary power analyses assuming a sample size of 500 observations (i.e., survivors) has found, for example, that this analytic sample will be sufficient to detect a correlation as small as 0.13 and a two-sample mean bivariate difference as small as 0.25 with a standard significance level (*α* = .05), equating to “small” effect sizes according to accepted standards [36].

### Participant involvement

The study’s participant involvement will be conceptualized as the HT survivors participating in the interviews and the S-EAG. During the interviews, survivors will be asked about their preferences for participating in research and ideas to increase research engagement among survivors. For example, survivors will be asked about their preferred study incentives and method for participation (e.g., phone interview, online survey), as well as strategies to address barriers to participation and questions that researchers should ask to learn about the effectiveness of an anti-trafficking program. In addition, survivors will be encouraged to invite other ATP clients to participate in the interviews and the S-EAG.

As noted above, members of the S-EAG will be involved in the study from its early stages through to the dissemination of findings in order to center survivors’ expertise and lived experiences in the study’s work. S-EAG members’ input during meetings will influence formulation of research questions, refinement of recruitment strategies, interpretation of outcomes, and dissemination of findings. Members’ voices will also be crucial towards infusing a focus on equity and justice throughout the study. S-EAG members will not actively participate in any of the study activities beyond the S-EAG and interviews and will not be listed on any internal (e.g., human subjects approval) or external (e.g., published manuscripts) products. However, the study team will note in such products the general participation of survivors in the study. All participant involvement will be reported in relevant products (i.e., manuscripts and reports) following the short-form version of the Guidance for Reporting Involvement of Patients and the Public [37].

## Discussion

All aspects of the study will also be guided by well-established guidelines for research with violence survivors, including: (a) do no harm; (b) identify and assess risk; (c) ensure confidentiality; (d) obtain voluntary and informed consent; (e) ensure privacy, safety, and security; (f) provide appropriate referral information and do not make promises that cannot be carried out; (g) be respectful and non-judgmental; (h) ask questions in a sensitive way; and (i) center issues of social justice and equity [20, 21, 38].

### Dissemination

Ninety days prior to the completion of the project, the study’s data and data management files (i.e., Stata syntax and the interview guides) will be submitted to the digital repository of the National Archive of Criminal Justice Data (NACJD), which will provide long-term access of the data to the research community, practitioners, and policymakers. Before submission to the NACJD for evaluation, all data will be de-identified (e.g., identifying information will be redacted from interview and focus group transcriptions). To adhere to the NACJD’s guidelines, the research team will develop a data archiving plan.

To the best of the team’s knowledge, this study will be the first known example of applying the EA methodology to an anti-trafficking program, making it a novel contribution to anti-trafficking research. Dissemination of findings will be accomplished through a variety of products and communication strategies for various practice-, research-, and policy-centric audiences to maximize impact of the potentially important implications. Overall, the study team will focus on developing dissemination products tailored for both practice professionals (e.g., final report, guides, pamphlets, infographics, white papers) and researchers (e.g., peer-reviewed manuscripts, refereed conference presentations). In addition, the team will publicize key project’s findings that may be of interest to the broader public through collaboration with public relations offices at both practice and research organizations to develop media stories (e.g., press releases, social media content) and to disseminate the products across a variety of settings, contexts, and channels.

## Conclusion

This study aims to conduct an evaluability assessment of a prominent anti-trafficking program in the United States. Using a systematic process, the study team will determine feasible evaluation strategies for ATP that will provide useful information to both improve ATP services at SH, and to build evidence to be applied to trafficking survivor services broadly. Thus, results from the EA have strong potential to develop robust evidence and products that can guide service provision not only for the specific organization under study, but also for other anti-trafficking interventions and service initiatives globally. Likewise, rigorous evaluation of anti-HT programs is crucial to ensuring appropriate service delivery proximally, and distally to work towards addressing trafficking survivors’ legal and immigration needs, ensuring their safe and permanent housing, establishing their fair and living-wage employment, as well as promoting their physical and mental health. Accordingly, while this EA study is underway, this protocol offers the field suggestions for how evaluations of anti-trafficking programs may be carried out, which in turn, may hopefully inform other, similar research efforts in the near future.

## Data Availability

Study data will be submitted to the digital repository of the National Archive of Criminal Justice Data per conditions of the external funder. This archive will provide long-term access of the data.

## Abbreviations

HT: Human trafficking
SH: Safe Horizon
ATP: Anti-Trafficking Program
EA: Evaluability assessment
S-EAG: Survivor Expert Advisory Group
I-EAG: Interdisciplinary Expert Advisory Group
NACJD: National Archive of Criminal Justice Data

## References

[1] Oram S, Stöckl H, Busza J, Howard LM, Zimmerman C (2012). Prevalence and risk of violence and the physical, mental, and sexual health problems associated with human trafficking: systematic review. PLoS Med.

[2] Rothman EF, Stoklosa H, Baldwin SB, Chisolm-Straker M, Kato Price R, Atkinson HG, HEAL Trafficking (2017). Public health research priorities to address US human trafficking. AJPH.

[3] Zimmerman C, Hossain M, Watts C (2011). Human trafficking and health: a conceptual model to inform policy, intervention and research. Soc Sci Med.

[4] Zimmerman C, Kiss L (2017). Human trafficking and exploitation: a global health concern. PLoS Med.

[5] Abas M, Ostrovschi NV, Prince M (2013). Risk factors for mental disorders in women survivors of human trafficking: a historical cohort study. BMC Psychiatry.

[6] Cannon AC, Arcara J, Graham LM (2018). Trafficking and health: a systematic review of research methods. Trauma Violence Abuse.

[7] Dell NA, Maynard BR, Born KR (2019). Helping survivors of human trafficking: a systematic review of exit and postexit interventions. Trauma Violence Abuse.

[8] Hom KA, Woods SJ (2013). Trauma and its aftermath for commercially sexually exploited women as told by front-line service providers. Issues Ment Health Nurs.

[9] Muftić LR, Finn MA (2013). Health outcomes among women trafficked for sex in the United States: a closer look. J Interpers Violence.

[10] Braveman P, Gruskin S (2003). Defining equity in health. J Epidemiol Community Health.

[11] Braveman P, Egerter S, Williams DR (2011). The social determinants of health: coming of age. Annu Rev Public Health.

[12] Macy RJ, Johns N (2011). Aftercare services for international sex trafficking survivors: informing US service and program development in an emerging practice area. Trauma Violence Abuse.

[13] Kaufman MR, Crawford M (2011). Research and activism review: sex trafficking in Nepal: a review of intervention and prevention programs. Violence Against Women.

[14] Menon B, Stoklosa H, Van Dommelen K (2018). Informing human trafficking clinical care through two systematic reviews on sexual assault and intimate partner violence. Trauma Violence Abuse.

[15] Davy D (2015). Understanding the support needs of human-trafficking victims: a review of three human-trafficking program evaluations. J Hum Traffick.

[16] Graham LM, Macy RJ, Eckhardt A (2019). Measures for evaluating sex trafficking aftercare and support services: a systematic review and resource compilation. Aggress Violent Behav.

[17] Davies R, Payne L (2015). Evaluability assessments: reflections on a review of the literature. Evaluation..

[18] Leviton LC, Khan LK, Rog D, Dawkins N, Cotton D (2010). Evaluability assessment to improve public health policies, programs, and practices. Annu Rev Public Health.

[19] Trevisan MS, Walser TM (2014). Evaluability assessment: improving evaluation quality and use.

[20] IOM. The IOM handbook on direct assistance for victims of trafficking. Geneva: International Organization for Migration; 2007.

[21] Zimmerman C, Watts C. World Health Organization ethical and safety recommendations for interviewing trafficked women. Geneva: World Health Organization; 2003.

[22] Trevisan MS (2007). Evaluability assessment from 1986 to 2006. Am J Eval.

[23] Rothman EF, Preis SR, Bright K (2020). A longitudinal evaluation of a survivor-mentor program for child survivors of sex trafficking in the United States. Child Abuse Negl.

[24] Ammerman A, Smith TW, Calancie L (2014). Practice-based evidence in public health: improving reach, relevance, and results. Annu Rev Public Health.

[25] Thurston WE, Potvin L (2003). Evaluability assessment: a tool for incorporating evaluation in social change programmes. Evaluation..

[26] Fraser MW, Galinsky MJ (2010). Steps in intervention research: designing and developing social programs. Res Soc Work Pract.

[27] Wight D, Wimbush E, Jepson R (2016). Six steps in quality intervention development (6SQuID). J Epidemiol Community Health.

[28] Carroll KM, Nuro KF (2002). One size cannot fit all: a stage model for psychotherapy manual development. Clin Psychol.

[29] Damschroder LJ, Aron DC, Keith RE (2009). Fostering implementation of health services research findings into practice: a consolidated framework for advancing implementation science. Implement Sci.

[30] Kulkarni SJ, Stylianou AM, Wood L (2019). Successful rules reduction implementation process in domestic violence shelters: from vision to practice. Soc Work.

[31] Macy RJ, Ermentrout DM, Redmond PH (2015). From novel to empirical: developing community-based programs into research-ready programs. Child Welfare.

[32] Rizo CF, Reynolds A, Macy RJ (2016). Parenting and safety program for system-involved female survivors of intimate partner violence: a qualitative follow-up study. J Fam Violence.

[33] Rizo CF, Wretman CJ, Macy RJ (2018). A novel intervention for system-involved female intimate partner violence survivors: changes in mental health. Am J Orthop.

[34] Stylianou AM, Counselman-Carpenter E, Redcay A. “My sister is the one that made me stay above water”: how social supports are maintained and strained when survivors of intimate partner violence reside in emergency shelter programs. J Interpers Violence Published Online First. 2018. 10.1177/0886260518816320. 10.1177/0886260518816320 30526216

[35] Wretman CJ, Rizo CF, Macy RJ (2019). A novel intervention for system-involved intimate partner violence survivors: changes in parenting. Res Soc Work Pract.

[36] Cohen J (1988). Statistical power analysis for the behavioral sciences.

[37] Staniszewska S, Brett J, Simera I, et al. GRIPP2 reporting checklists: tools to improve reporting of patient and public involvement in research. BMJ. 2017;358:j3453. https://www.bmj.com/content/358/bmj.j3453. 10.1136/bmj.j3453 PMC5539518 28768629

[38] Cannon AC, Arcara J, Arnoff E (2014). Trafficking in persons and health: a compendium of monitoring and evaluation indicators.

